# Poster Session II - A250 REAL-WORLD EFFECTIVENESS, SAFETY, AND DOSE MODIFICATIONS OF UPADACITINIB IN PATIENTS WITH CROHN’S DISEASE: A CANADIAN MULTICENTER STUDY

**DOI:** 10.1093/jcag/gwaf042.250

**Published:** 2026-02-13

**Authors:** L M van Lierop, R Dang, T Omeltchenko, M Adriaanse, M Gozdzik, K Kroeker, B Halloran, K Wong, F Peerani, J H Chow, J McCurdy, N Narula, J Siffledeen, L Du, T Dang, C Ma, F Hoentjen

**Affiliations:** Gastroenterology, University of Alberta Faculty of Medicine & Dentistry, Edmonton, AB, Canada; McMaster University, Hamilton, ON, Canada; Gastroenterology, University of Alberta Faculty of Medicine & Dentistry, Edmonton, AB, Canada; Gastroenterology, University of Alberta Faculty of Medicine & Dentistry, Edmonton, AB, Canada; Gastroenterology, University of Alberta Faculty of Medicine & Dentistry, Edmonton, AB, Canada; Gastroenterology, University of Alberta Faculty of Medicine & Dentistry, Edmonton, AB, Canada; Gastroenterology, University of Alberta Faculty of Medicine & Dentistry, Edmonton, AB, Canada; Gastroenterology, University of Alberta Faculty of Medicine & Dentistry, Edmonton, AB, Canada; Gastroenterology, University of Alberta Faculty of Medicine & Dentistry, Edmonton, AB, Canada; University of Ottawa Faculty of Medicine, Ottawa, ON, Canada; University of Ottawa Faculty of Medicine, Ottawa, ON, Canada; McMaster University, Hamilton, ON, Canada; Gastroenterology, University of Alberta Faculty of Medicine & Dentistry, Edmonton, AB, Canada; Gastroenterology, University of Alberta Faculty of Medicine & Dentistry, Edmonton, AB, Canada; Gastroenterology, University of Alberta Faculty of Medicine & Dentistry, Edmonton, AB, Canada; University of Calgary Cumming School of Medicine, Calgary, AB, Canada; Gastroenterology, University of Alberta Faculty of Medicine & Dentistry, Edmonton, AB, Canada

## Abstract

**Background:**

Upadacitinib (UPA) is widely used for the treatment of Crohn’s disease (CD) but data on clinical outcomes in daily practice remain scarce.

**Aims:**

To assess the real-world effectiveness, safety, and dose modifications of UPA in patients with CD.

**Methods:**

This multicenter retrospective cohort study included adults with CD initiating UPA at 7 Canadian centers (2019/10–2025/04). Patients were followed from treatment start until database lock (June 2025), discontinuation, or loss to follow-up (censored). The primary endpoint was corticosteroid-free clinical remission (CFCR; HBI <5 off systemic corticosteroids ≥90 days). Biochemical (FCP <250 µg/g and/or CRP <10 mg/L) and endoscopic (SES-CD ≤4) remission rates were captured. We documented adverse events (AEs), analyzed drug persistence by Kaplan–Meier, and evaluated predictors of CFCR by Cox regression.

**Results:**

We included 295 patients (mean age 44.9 ± 15.9 years; 46.1% female) with a median follow-up of 37.7 weeks (IQR 42.4). During the maintenance phase, 24 (8.1%) patients underwent dose escalation. Prior exposure to any and ≥3 advanced therapies was noted in 93.2% (n = 275) and 39.3% (n = 116), respectively, and 25.1% (n = 74) used corticosteroids at baseline. UPA starting doses were 15 mg in 6.4% (n = 19), 30 mg in 8.1% (n = 24), and 45 mg in 84.4% (n = 249) of patients. At the end of follow-up, 55.3% (n = 163) achieved CFCR; clinical and biochemical remission rates were 60.0% (n = 177) and 44.4% (n = 131). CFCR was achieved in 40.0% (n = 8), 63.5% (n = 47), 61.9% (n = 52), and 48.3% (n = 56) of patients exposed to 0, 1, 2, or ≥ 3 advanced therapies, respectively. Endoscopic remission occurred in 24/61 colonoscopies. There were 145 AEs reported in 104 patients, predominantly acne (n = 28), and infections (n = 29; 8 classified as serious (S)AEs). Of all AEs, 10.3% were SAEs, including thrombo-embolic events (n = 2), herpes zoster (n = 1), and malignancy (n = 1). There were 29 (9.8%) patients who experienced 40 CD-related hospitalizations, and 26 (8.8%) patients underwent 35 surgeries: 12 intestinal resections, 3 colectomies, 16 perianal, and 4 other IBD-related procedures. UPA was discontinued in 19.3% (n = 57), mainly for lack of efficacy (n = 30) or AEs (n = 13). Drug persistence at year 1 was 73.8%. No factors were significantly associated with CFCR on Cox regression.

**Conclusions:**

In this large and refractory Canadian CD cohort with extensive prior exposure to advanced IBD therapies, a substantial proportion of patients experienced clinical benefit from UPA. Treatment persistence was high while SAEs were rare, and AEs resulted in treatment discontinuation in < 5%.

**Funding Agencies:**

None

